# Effect of *Arum rupicola* Boiss *rupicola* Extracts on Visceral Larva Migrans in Mice

**DOI:** 10.1007/s11686-024-00970-4

**Published:** 2025-01-24

**Authors:** Gozde Nur Akkus, Tugrul Atalay, Sinem Akdeniz, Husamettin Ekici, Dincer Yildiz, I. Safa Gurcan, Kader Yildiz

**Affiliations:** 1 Health Sciences Institute, Department of Parasitology, Kirikkale University, Kirikkale, 71450 Türkiye; 2 Sefaatli Vocational School, Yozgat Bozok University, Yozgat, 66800 Türkiye; 3 Faculty of Veterinary Medicine, Department of Pharmacology and Toxicology, Kirikkale University, Kirikkale, 71450 Türkiye; 4 Faculty of Veterinary Medicine, Department of Anatomy, Kirikkale University, Kirikkale, 71450 Türkiye; 5 Faculty of Veterinary Medicine, Department of Bioistatistic, Ankara University, Ankara, 06110 Türkiye; 6 Faculty of Veterinary Medicine, Department of Parasitology, Kirikkale University, Kirikkale, 71450 Türkiye

**Keywords:** *Toxocara canis*, Larva, Mice, Migration, *Arum rupicola* Boiss *rupicola*, Antiparasitic activity

## Abstract

**Purpose:**

In the present study, the effects of leaf and rhizome extracts of *Arum rupicola* Boiss *rupicola* were searched on the infective stage *Toxocara canis* larvae (L3) in the experimentally infected mice.

**Methods:**

Four-six week-old male BALB/c mice were divided into eight groups (G1-8, each group consisted of 7 mice), and they were infected orally with 500 *T. canis* eggs with L3. After 24 h, the following treatment protocol was applied to the groups for five days: G-1: Albendazole (ABZ) (100 mg/kg), G-2: Leaf Extract (10 mg/mL) + ABZ (100 mg/kg), G-3: Leaf Extract (40 mg/mL) + ABZ (100 mg/kg), G-4: Rhizome Extract + ABZ (100 mg/kg), G-5: Leaf Extract (10 mg/mL), G-6: Leaf Extract (40 mg/mL), G-7: Rhizome extract, G-8: Negative control. The necropsy was performed on the 45th day of experimental infection.

**Results:**

The rhizome extract significantly reduced the effectiveness of ABZ compared to leaf extracts. The number of larvae in rhizome extract-treated mice (G7) was similar to ABZ-treated mice (G1) (*P* < 0.05). The larval number in mice administered leaf extract (10 mg/mL and 40 mg/mL) was slightly decreased. But no significant difference was detected in the larval number depending on the dose of leaf extracts (*P* > 0.05).

**Conclusion:**

The administration of the leaf and rhizome extracts did not contribute to the effectiveness of ABZ. Also the effect of ABZ on the larvae number obtained from the tissues was reduced by the rhizome extract. The larvae number in the group in which rhizome extract was given alone was almost close to the group in which ABZ was administered. The results provide insight for further research on the antiparasitic properties of *A. rupicola* Boiss *rupicola* rhizome extract.

## Introduction

Adult *Toxocara canis*, Nematoda: Ascarididae, lives in the intestines of dogs [1, 2]. The life cycle of the parasite is complex. Some animal species such as mice, pigs, sheep, rabbits, poultry, and even earthworms play a role as paratenic hosts in the life cycle [3]. Its infective stage larvae (L3) are found in various parenchymatous tissues and muscles of paratenic hosts [2, 3]. Due to the zoonotic features of the parasite, humans are also among the paratenic hosts [1].

Toxocariasis can be transmitted to humans through different ways, including by eating *T. canis* eggs after developing L3 or raw/undercooked paratenic host tissues containing L3 [2, 3]. In addition to *T. canis*, *Toxocara cati* is also responsible for toxocariasis in humans, and the serological tests used in the diagnosis of toxocariasis cannot distinguish infection due to these two parasite species. Toxocariasis occurs in four different forms in humans (visceral larva migrans, ocular toxocariasis, covert toxocariasis and neuro-toxocariasis) [4, 5]. Children are one of the risk groups for toxocariasis. They are at higher risk of being exposed to this parasite when playing in public parks or playgrounds that are likely contaminated with dog feces. In addition, habits such as eating soil, biting nails, or bringing fomits into the mouth also increase the risk of infection [6].

After infection, L3 release in the small intestine of paratenic hosts including humans and migrates to the liver, then to the lungs and other parenchymatous organs via the bloodstream and lymph vessels [1, 3]. Unlike the development in dogs, L3 does not become an adult parasite in paratenic hosts [2, 5]. Specific clinical signs are generally not observed during toxocariasis in the paratenic hosts [3]. However, serious illness and organ damage may occur due to the host inflammatory reaction developed against migrating larvae [4].

There is no approved treatment protocol for visceral larva migrans in humans. Drugs have limited effectiveness against visceral larva migrans in humans. Albendazole (ABZ) is preferred as the first option for toxocariasis treatment. The treatment regimen with this drug has not yet been standardised for toxocariasis. In Europe and the United States, ABZ is administered to both adults and children at a dose of 400 mg twice a day for 5 days. However, the clinical cure rate of this treatment protocol is approximately 32%. On the other hand, in Japan, ABZ is administered orally at a daily dose of 10–15 mg/kg for 4 weeks, followed by a break for 2 weeks, and then repeat the same protocol for the next 4 weeks, which is the same regime used for the treatment of cystic echinococcosis in humans [7, 8]. However, ABZ administered using this protocol may cause some side effects in patients. Alternative treatment options for support to anthelmintics are being investigated to alleviate the clinical signs. In particular, herbal extracts have been tested both for their treatment efficiency alone and for the potential to increase the effectiveness of the anthelmintics used in vivo [9–14].

*Arum rupicola* Boiss *rupicola* is a native plant in a wide geographical area, including Türkiye, Israel, Iran, Iraq, Lebanon, Syria, Palestine, Georgia and Azerbaijan [15]. The plant leaves begin to appear in spring, and the flowering time varies depending on the region but is generally in March-April. The plant has a rhizome under the soil. Its round fruits are light green in summer, and they ripen and turn orange-red in autumn [16]. Arum plants are gathered for food by local people living in Caucasus, Türkiye, Iran and Bulgaria [15]. Moreover, these plants are used in traditional medicine for recovery from cancer, cought, haemorrhoid, headache, and anticestodicidal in different geographical areas [17–22]. Some pharmacological activities such as antimicrobial, antifungal, antioxidant, and anti-inflammatory activities of *Arum* plants are known [15, 23–26]. Some cytotoxic effects of *A. rupicola* were observed on adenocarcinoma cells in vitro and breast cancer cells [20, 27]. There is some information on the antiparasitic effects of *Arum* plants [15, 28, 29]. The plant known as “gigarum”— identified as *Arum italicum*—was utilised by the Etruscans to cure *Fasciola hepatica* [30]. *Arum maculatum* was used to treat malaria and it was mentioned among the Renaissance herbals [15]. *Arum maculatum* rhizomes have an insecticidal effect on some plant lice species [28]. Only one report has been found on the antiparasitic effects of *A. rupicola* Boiss *rupicola*. In vitro larvicidal activities of the ethanolic leaf extract and the aqueous rhizome extract of *A. rupicola* Boiss *rupicola* on *T. canis* L3 are reported [29]. The paralysed L3 rate is found to be higher in the leaf extract-applied groups than in the rhizome extract-applied groups in vitro [29].

Mouse models are often preferred for studies on VLM because of the similarity of migration and pathogenesis between mice and humans [31]. The BALB/c mouse is the most suitable animal model for toxocariasis [32]. To detect in vivo antiparasitic efficiency of the ethanolic extract of *A. rupicola* Boiss *rupicola* leaves and the aqueous extract of its rhizome part on *T. canis* larvae, the extracts were administered to experimentally infected mice. Treatment efficiency of the extracts on *T. canis* L3 in mice was investigated both when it is applied alone and for the potential to increase effectiveness enhanced of ABZ.

## Materials and Methods

### The Extracts of *Arum rupicola* Boiss *rupicola*

*Arum rupicola* Boiss *rupicola* was collected from Karaçakıl Locality of Güdül District, Ankara, Türkiye for a previous study on the in vitro efficiency of the plant extracts on *T. canis* L3 and eggs [29]. The leaves and rhizome extracts prepared for an in vitro study [29] were used. Briefly, the leaf parts were extracted with 50% ethyl alcohol, the extract was pulverized in a vacuum evaporator, and the crude was stored at -80 °C in a sterile microcentrifuge tube. The rhizome parts were washed and the outer membrane-like shell was removed. After weighed, it was homogenised in sterile distilled water (5 mL distilled water per gram rhizome). The homogenate was then placed in a shaker (Boeco TS-100 C) and kept at 390 rpm at 4 °C for 60 min, filtered through cheesecloth, portioned into a sterile cryotube and stored at -80 °C. The treatment doses were selected based on cytotoxic potential and haemolytic activity of the extracts [29]. The ethanolic extract of leaf has cytotoxic potential and haemolytic activity at a 10 mg/mL dilution, whereas the aqueous extract of rhizome does not have these effects even if undiluting [29]. In the present study, the leaf extract was used at 10 and 40 mg/mL dilutions and the crude leaf extract was diluted with RPMI-1640 before use and the aqueous rhizome extract was used undiluted.

### Gas Chromatography-Mass Spectrometry (GC-MS) Analysis of the Extracts

The leaf and rhizome extracts were dissolved in methanol. Then, the fatty acid content of the A. rupicola Boiss rupicola extracts was determined using GC-MS analysis with Shimadzu GCMS/QP2010 ultra with column Rtx-5MS (30 m×0.25 mm) 0.25 μm thickness. Helium gas was used as a carrier. Analytical conditions were 1 µL extract sample in methanol extract GC oven temperature was programmed to 40–270 °C. The leaf and rhizome extracts were injected at 280.0 °C, the column oven temperature was kept at 40 °C, the split ratio was 1.0, the pressure was maintained at 112.0 kPa, and column flow was 2 mL/min. The total program time was 121 min. GC-MS analyses were performed on detector GCMS-QP2010 ion source temperature was kept at 300 °C.

### Developing *T. canis* L3 in the Eggs

Adult *T. canis* brought to veterinary clinics by dog owners were examined under a stereomicroscope, and the female parasites were washed three times with sterile distilled water. The uterus was dissected carefully, and the parasite eggs were washed with sterile distilled water. The eggs were placed in 0.05% formalin solution and placed in an incubator (28 °C). The eggs were incubated for four weeks in this condition and examined daily for larval development using a light microscope [33]. After completing 4 weeks-incubation, the eggs were washed three times in sterile distilled water to remove the formalin solution and once in RPMI-1640 (x300 g, 5 min). Motile larva was observed in *T. canis* eggs. The egg number was counted and the suspension was adjusted with RPMI-1640 as 500 eggs per 0.2 mL, the volume that could be administered to a mouse.

### Experimental Infections of Mice and Treatment

Four to six-week-old male BALB/c mice were used in the study [32]. The mice were orally inoculated with *T. canis* eggs with L3 (500 eggs/0.2 mL) by intra-gastric gavage [34] The egg suspension was pipetted carefully before being sampled for each mouse infection. Following the infection, the mice were monitored for vomiting, and 24 h later, the feces were collected from the cages and examined for *T. canis* eggs and larvae by centrifugal flotation and the Baerman method, respectively. The fecal examination was repeated for 3 days after the experimental infection.

Eight experiment groups (G) each of them consisted of 7 mice were composed as follows: G-1: Only ABZ, G-2: Leaf extract (10 mg/mL) + ABZ, G-3: Leaf extract (40 mg/mL) + ABZ, G-4: Rhizome extract + ABZ, G-5: Leaf extract (10 mg/mL), G-6: Leaf extract (40 mg/mL), G-7: Rhizome extract (undiluted) G-8: Negative control (infected-untreated). ABZ was administered as 100 mg/kg dose per mouse [33]. The treatment protocols started 24 h after the experimental infection and continued for 5 consecutive days [12]. The treatment was applied at the same time every day. ABZ was administered firstly and the extract was administered one hour later in the mice (G-2, G-3 and G-4). 0.9% isotonic NaCl_2_ solution (0.2 mL per mouse) was administered to the control group (G-8) by intra-gastric gavage for 5 consecutive days.

### Necropsy and Detection of L3 in Mouse Tissues

After completion of the treatment period, the mice were fed ad libitum and observed daily for 45 days. Then the mice were sacrificed with cervical dislocation. The brain, liver, lung, heart, spleen, kidney, and muscles (intercostal, front and back leg) were dissected. Each organ (except brain tissue) was weighed and recorded. Brain tissue was pressed on the slide and examined under a light microscope. The other tissues were placed in separate sterile Petri dishes with lids, trimmed with scissors, and then added from the pepsin-HCl solution (5 g pepsin + 7 mL HCl + 988 mL 0,9% saline solution). The ratio of tissue to pepsin-HCl solution was approximately 1:10 (g: mL) [35]. After incubation at 37 °C for 2 h, the lysates were sieved into sterile tubes and centrifuged at 2000 rpm for 10 min. The supernatant was discarded, and the sediment was washed with sterile saline solution [36]. All sediments were examined under a light microscope for the presence of L3. The sediments not examined during the necropsy day were placed in 2% formalin solution and stored in the refrigerator (+ 4 °C).

### Statistical Analysis

In this study, power analysis was applied to determine the minimum sample size and to avoid Type II error. Also with appropriate power is more likely to produce reliable, reproducible results. With an effect size (d) of 0.55, alpha (α) of 0.05 and power (1-β) of 0.80, the minimum sample size (n) was determined as 56. Differences between groups were investigated with one-way ANOVA analysis, and results with a P value less than 0.05 were considered significant. Statistical analysis of the results was performed using SPSS 15.01 (SPSS Inc., Chicago, IL, USA).

## Results

### Fatty Acid Contents of the Extracts

The GC-MS analysis of the rhizome extract revealed the presence of five fatty acids as compounds with the highest abundance. These compounds were: octadecanoic acid 2 3-dihydroxypropyl ester, octadecanoic acid, methyl ester, 9-octadecenoic acid(Z)-, methyl ester, hexadecanoic acid and tridecanoic acid, methyl ester. The GC-MS analysis of the leaf extract revealed the presence of three fatty acids as compounds with the highest abundance. These compounds were: 7-hexadecenoic acid, methyl ester, 2-propenoic acid, methyl ester and 1-propyl methyl ether (Table 1).

**Table 1:** The fatty acid contents of the leaf and rhizome extracts of *A. rupicola* Boiss *rupicola*

Extract	Fatty acid	RT	Area %	Formula	Molecular weight
Leaf	2-Propenoic acid, methyl ester (CAS)	4.220	4.43	C_4_H_6_O_2_	86.0892
	7-Hexadecenoic acid, methyl ester, (Z)-	80.518	1.37	C_17_H_32_O_2_	268.4348
Rhizome	Octadecanoicacid 2,3-dihydroxypropyl ester	107.479	56.27	C_21_H_42_O_4_	358.5558
	Hexadecanoic acid (CAS)	76.018	25.72	C_16_H_32_O_2_	256.4241
	Tridecanoic acid, methyl ester	72.135	8.03	C_14_H_28_O_2_	228.3709
	9-Octadecenoic acid (Z)-, methyl ester	80.562	4.54	C_19_H_36_O_2_	296.4879
	Octadecanoic acid, methyl ester (CAS)	81.841	3.56	C_19_H_38_O_2_	298.5038

*Abbreviation* RT: Retention time

Formula and molecular weight of fatty acids were obtained from National Institute of Standards and Technology (https://www.nist.gov, Accession:10.10.2024)

### Experimental Infections of Mice and Treatment

**Fig. 1:**
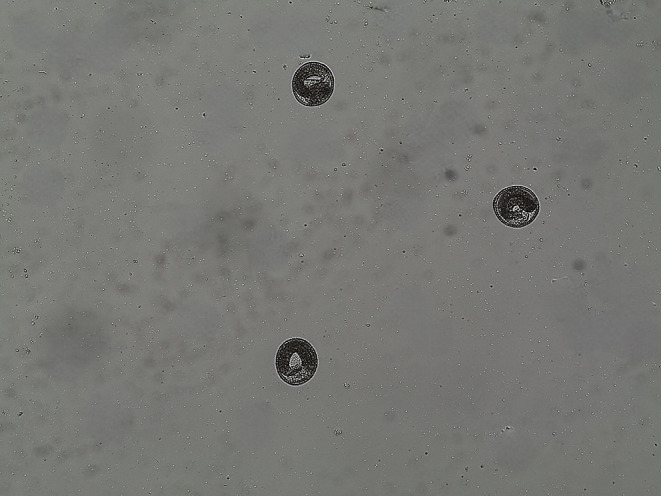
*Toxocara canis* eggs with the infective stage larvae after 28 days of incubation (x10)

After inoculation with the *T. canis* eggs eggs included L3 (Fig. 1) by intra-gastric gavage, vomiting was not observed in the mice. No *T. canis* eggs or larvae were detected in the feces of mice. Vomiting was not observed in the mice after administration ABZ or the extracts. The extracts were well tolerated by the mice without any clinical signs. All infected mice were alive and active throughout the 45-days of the experiment.

### Necropsy and Detection of L3 in Mouse Tissues

**Fig. 2:**
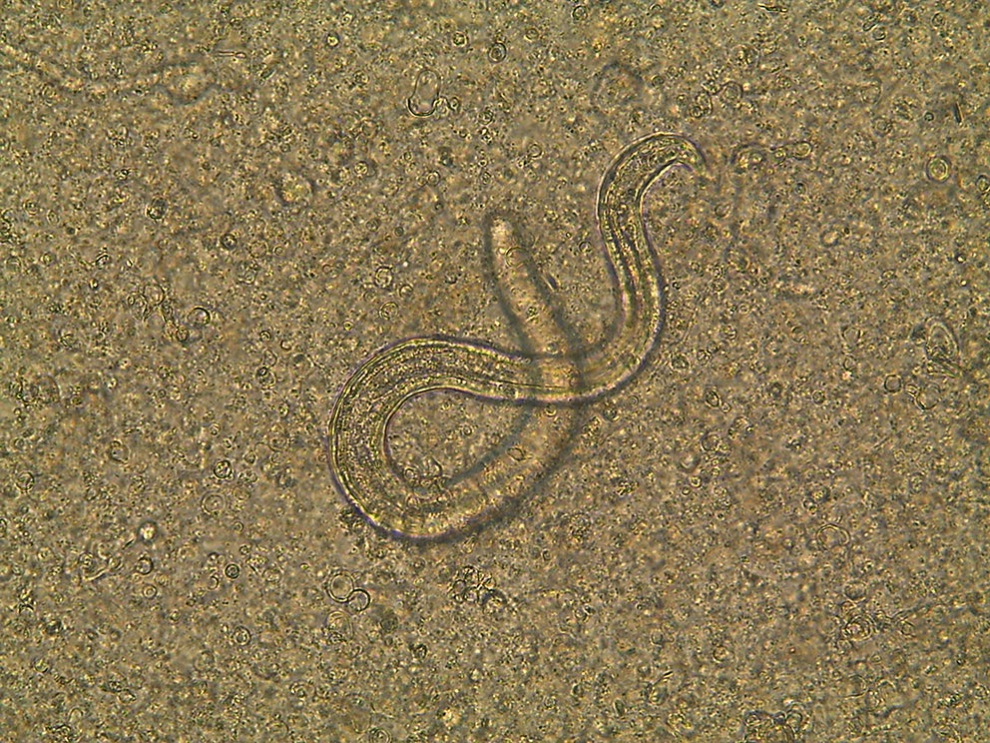
Motile *T. canis* larvae in brain tissue of mouse (x40)

**Fig. 3:**
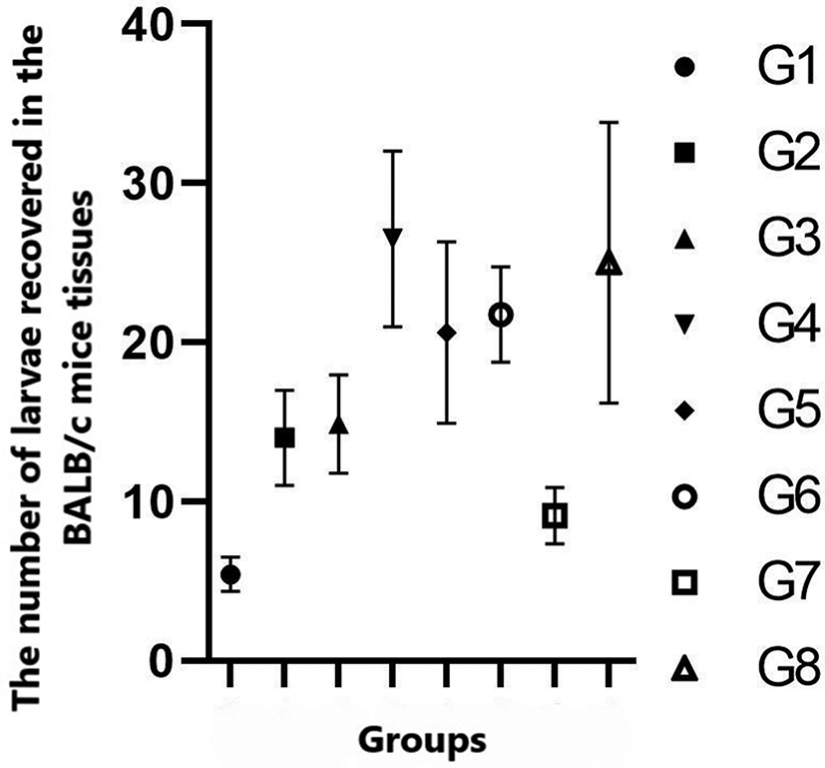
The mean larvae number of the groups (G). G1: Albendazole (ABZ) (100 mg/kg), G2: Leaf extract (10 mg/mL) + ABZ (100 mg/kg), G3: Leaf extract (40 mg/mL) + ABZ (100 mg/kg), G4: Rhizome extract + ABZ (100 mg/kg), G5: Leaf extract (10 mg/mL), G6: Leaf extract (40 mg/mL), G7: Rhizome extract, G8: Negative control

During necropsies, motile larvae were observed in brain tissues (Fig. 2). The larvae were also detected in the other parenchymatous organs and muscles of the mice. The mean larva number of the groups is indicated in Fig. 3. The number of larvae obtained from the untreated group (G-8) was set as 100%. The number of larvae decreased significantly in mice administered only ABZ (G-1) (78.28%) compared with the untreated group (G-8) (*P* < 0.05). In comparison to the untreated group, larval numbers decreased in the groups (44%, 40.48%, 0%, 17.48%, 66% and 63.48 in the G-2, G-3, G-4, G-5, G-6 and G-7, respectively). The leaf (10 mg/kg and 40 mg/kg) and rhizome extracts decreased the effectiveness of ABZ on L3 in mice administered together (44%, 40.48% and 0% in the G-2, 3 and 4, respectively). In particular, the rhizome extract significantly reduced the effectiveness of ABZ administered together (G-4) compared with leaf extracts (G-2 and 3) (*P* < 0.05). The number of larvae in the tissues of mice administered only the rhizome extract (G-7) decreased nearly as G-1 (63.48% vs. 78.28%) (*P* < 0.05). However, larval numbers in mice administered ABZ and the rhizome extract (G-4) were similar to those in the untreated group (G-8).

**Fig. 4:**
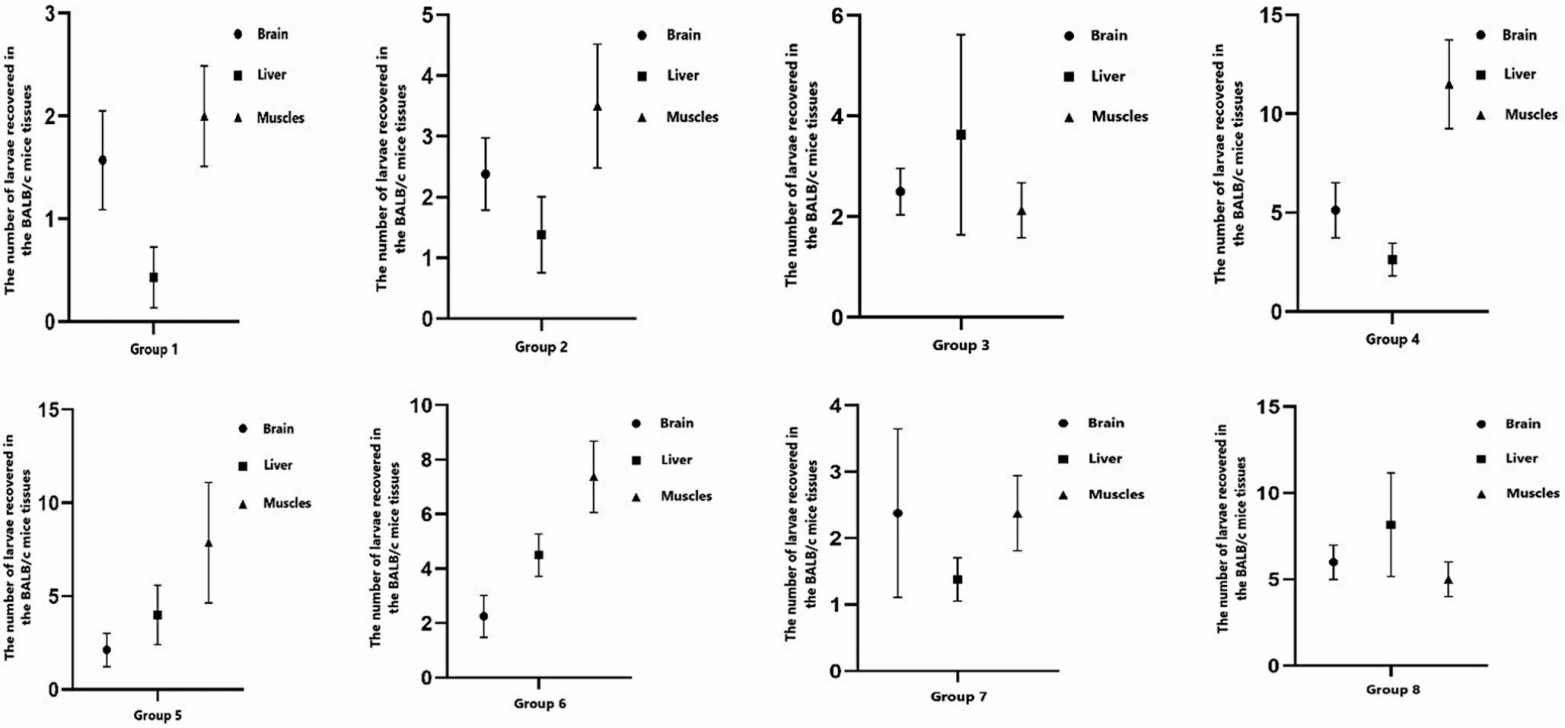
The average number of larvae in the tissues of necropsied mice. Group 1: Albendazole (ABZ) (100 mg/kg), Group 2: Leaf extract (10 mg/mL) + ABZ (100 mg/kg), Group 3: Leaf extract (40 mg/mL) + ABZ (100 mg/kg), Group 4: Rhizome extract + ABZ (100 mg/kg), G5: Leaf extract (10 mg/mL), G6: Leaf extract (40 mg/mL), G7: Rhizome extract, G8: Negative control

The number of larvae was slightly decreased in mice (44% and 40.48%) administered two leaf extract doses (10 mg/mL and 40 mg/mL). No significant difference was detected in larval number according to leaf extract dose (*P* > 0.05). The number of larvae was reduced in the tissues of mice administered leaf extracts together with ABZ (44% and 40.48% in the G-2 and G-3, respectively) compared with that in the tissues of mice administered only leaf extracts (17.48% and 66% in the G-5 and G-6, respectively) (*P* < 0.05). The average number of larvae in the tissues of necropsied mice is presented in Fig. 4. There was no statistical difference between the extract-administered mice regarding the number of larvae detected in the brain, liver, and muscles.

## Discussion

Herbal extracts have been studied for their ability to improve anthelmintics’ efficacy and treat symptoms on their own. Many studies have shown the in vitro efficacy of plant extracts on *T. canis* [29, 37–39]. However, the number of studies investigating the in vivo effectiveness of plant extracts is low [9–12] compared with that of in vitro studies [29, 37–39]. In vivo herbal efficiency studies on visceral larva migrans are designed differently from each other concerning the mouse strain used for experimental infection, number of *T. canis* eggs/larvae for infection, time to start administration of extracts, administration duration, and time to perform the necropsy [9–12]. Studies investigating the effects of herbal extracts have mostly focused on their therapeutic properties of the extracts on visceral larva migrans [9–12], whereas preventative effects of some herbal extracts have also been investigated [14]. Compound 17 (C17) obtained from *Picrasma quassioides* and *Ailanthus altissima* plants together with polyethylene + glycol + liposome have been administered between 28 and 36 th days of post-infection to BALB/c mice experimental infected with 300 invasive *T. canis* larvae [9]. The number of larvae in the brain and skeletal muscle decreased after C17 and *A. altissima* were administered together with polyethylene + glycol + liposome whereas the effect is not reported when C17 is administered alone in mice at the 50th day [9]. Ethanolic extract of *Cassia nodosa* has been administered once daily for 5 consecutive days starting on the first day of infection to BALB/c mice infected with 1000 *T. canis* eggs [10]. The extract of *C. nodosa* is more effective than ABZ in reducing the total number of larvae in the tissues of mice during necropsy at the 45th day of infection [10]. Hexane extract (30 mg/kg) and 10% infusions (1500 mg/kg) of *Chenopodium ambrosioides* and Nutridesintox, nutritional supplement included *Daucus carota L.*,* Cucurbita pepo L.*,* Allium sativum L. Sesamum indicum L. and Triticum aestivum L.*, have been administered daily between 10 and 12 day after infection to CD-1 mice infected with 300 *T. canis* eggs [13]. These treatments did not reduce the larvae burden in mice tissues on the 17th day post-infection [13]. In another study, *Nigella sativa* oil (0.15 mL) alone and together with ABZ (100 mg/kg) have been administered once daily for 5 consecutive days starting on the first day of post-infection to Swiss albino mice infected with 750 *T. canis* eggs [12]. It was observed that *N. sativa* oil increased the effectiveness of ABZ in the mice during necropsies (7, 14, 28, 45, and 60th days of the post-infection) [12]. Similarly, the number of larvae decreased after administration of *N. sativa* extract (100 mg/kg and 200 mg/kg) and ABZ (100 mg/kg) once daily for 7 consecutive days starting on the first day of post-infection to BALB/c mice infected with 500 *T. canis* eggs; however, this effect is not reported in the mice that administered only *N. sativa* extract [11]. In the present study, the leaf (10 mg/mL and 40 mg/mL) and rhizome extract of *A. rupicola* Boiss *rupicola* alone and together with ABZ were administered once daily for 5 consecutive days starting 24 h after infection of BALB/c mice with 500 *T. canis* eggs. The decrease in larval numbers in the group where rhizome extract was applied alone was found to be close to the ABZ-applied group 45th days of the post-infection. The rhizome and leaf extracts of *A. rupicola* Boiss *rupicola* reduced the effectiveness of ABZ against *T. canis* L3 in the experimentally infected mice.

The efficiency of some herbal extracts on visceral larvae migrans is also evaluated by histopathology and the changing of immune cells in infected mice [10–14]. Some authors have reported decreasing both pathological lesions and the number of larvae in tissues of infected mice after various extracts were administered [10–12, 14], on the contrary reductions in the inflammation are reported in mouse tissues without changed larvae number [13]. In the present study, the efficiency of *A. rupicola* Boiss *rupicola* leaf and rhizome extracts was not evaluated by histopathology and immunological parameters. The doses of *T. canis* eggs are different for developing VLM in mice experimentally [32]. The infectious dose below 500 *T. canis* eggs is accepted as a low dose and no significant pathological change is observed in the parenchymatous organs such as brain, lung, and liver in the mice experimentally infected by that dose. As the infection dose increases, the severity of the immune response also increases in the organism [40, 41]. In this study, the BALB/c mice were infected with 500 *T. canis* eggs because the effects of immune response on the experimental model were desired to be minimal.

Hepato-pulmonary and myotropic neurotropic phases are the two phases of migration route of *T. canis* L3 in paratenic hosts, including mice. During the first phase, the larvae reach the liver and lungs in the first week after infection, and after 48 h, most larvae are found in the liver, 4 days later in the lung and heart. The second phase occurs when the larvae move throughout the body and gather in the brain and muscles, the larvae reach the brain 14 days later [32]. In the present study, the plant extracts were administered five consecutive days to mice 24 h after the experimental infection to observe the efficiency on the hepato-pulmonary phase. The necropsies were performed on the 45th day of the experimental infection when the larvae possibly completed the myotropic neurotropic phase in the mice.

*Arum rupicola* extracts included various fatty acid content [15, 42]. In the present study, fatty acids were detected in the leaf and rhizome extracts of *A. rupicola* Boiss *rupicola.* Some of them (hexadecanoic acid, octadecanoic acid and tridecanoic acid) were saturated fatty acids. Some antiparasitic effects are reported from saturated fatty acids to *Giardia intestinalis* in vitro [43]. The rhizome of the *Arum* plant contains lectins [44]. Lectins are measured by liquid chromatography due to its non-volatile properties [45]. Before this process, the lectins must be purified and dialyzed from the extract. Similarly, genistein is a non-volatile flavonoid. It also measures by liquid chromatography. *Arum maculatum* rhizomes have an insecticidal effect on *Lipaphis erysimi* and *Aphis craccivora* (lice species) parasitised on the leaves of plants, and this effect develops after the rhizome lectin binds to the intestinal epithelium of the lice [28]. *Arum rupicola* Boiss *rupicola* rhizome extract has a larvicidal effect on infective stage *T. canis* larvae in vitro [29]. In this study, the rhizome extract showed a similar effect to that of ABZ in terms of the number of larvae in mice tissues (*P* < 0.05). However, the effect was not observed in mice treated with rhizome extract along with ABZ. Moreover, the rhizome extract reduced the activity of ABZ on *T. canis* larvae in the mice tissues. Some foods and drugs can interact in the organism. The interaction could change the pharmacokinetics and/or pharmacodynamics of the drug. Sometimes undesirable consequences such as therapeutic insufficiency and drug toxicity may develop in the organism [46]. In the present study, the reason why ABZ applied with rhizome extract was not effective on *T. canis* L3 in mice is not fully known. Commercial ABZ suspension was administered to the mice in this study. This suspension contains some excipients such as microcrystalline cellulose, sucrose, glycerine, methyl parahydroxy benzoate, propyl parahydroxy benzoate, carboxy methyl cellulose sodium, saccharine sodium. Lectins are (glyco) proteins and interact reversibly with carbohydrates [15]. It was thought that the rhizome extract may have interacted with these excipients found in commercial ABZ suspension and thus reducing the effect of ABZ on L3 in the mice.

The leaves of the *Arum rupicola* have some isoflavonoids such as genistein, genistein 8–C-glucoside and orobol [27]. Genistein is primarily responsible for the paralysis of helminths [47]. In an in vitro study, the leaf extract of *A. rupicola* Boiss *rupicola* causes paralysis on infective stage *T. canis* larvae [29]. In the present study, the total number of larvae in mice administered the leaf extract alone decreased slightly. We did not know the reason for the different effects observed in vivo and in vitro experiments using *A. rupicola* Boiss *rupicola* leaf extract. Many reasons can be responsible for these differences. Plants metabolize in the organism, many chemical mechanisms and enzymatic pathways play an important role in the process of metabolizing and absorbing of the plant metabolites in the organism. The metabolites show different effects on the organism. Moreover, many host-dependent factors can affect their bioavailability. In this study, the effects of *A. rupicola* Boiss *rupicola* extracts on *T. canis* L3 during in vivo experiments are different from the results of the previous in vitro experiment [29]. In a previous experiment, larvicidal activity is reported in both the ethanolic leaf extract and the aqueous rhizome extract of *A. rupicola* Boiss *rupicola* on *T. canis* L3 after hatching from parasite eggs [29]. The paralysed *T. canis* L3 rate is higher in the groups applied leaf extract than in the rhizome extract [29]. In the present study, the number *T. canis* L3 in the tissues of mice administered only the rhizome extract was similar to ABZ administered group (*P* < 0.05), however, the L3 number was a slight decrease in the leaf extracts administered groups. The different effects may have been observed in vivo experiments due to the bioactivity of the metabolites of the leaf extract of *A. rupicola* Boiss *rupicola*.

Several animal species eat some plants to help with digestion or eradicate parasites, bacteria and viruses in animals [48]. The rhizome parts of *A. rupicola* Boiss *rupicola* are consumed by wild pigs after being exhumed from the soil in nature [29]. Aqueous rhizome extract of *A. rupicola* Boiss *rupicola* has no cytotoxic potential on the L929 mouse fibroblast cell line whereas the cytotoxicity activity is reported at 10 mg/mL dilution of the ethanolic leaf extract [29]. Similarly, haemolytic activity is only reported from the ethanolic leaf extract [29]. Pigs are one of the paratenic hosts in the *T. canis* biology [3]. Considering the effects of the rhizome extracts on the decreasing of *T. canis* L3 in mice tissues in the present study, wild pigs may be eating the rhizomes in nature to decrease *T. canis* L3 larvae in their tissues.

## Conclusions

Alternative treatment options for support to anthelmintics are being investigated in visceral larvae migrans models. In this study, the administration of the leaf and rhizome extracts of *A. rupicola* Boiss *rupicola* together with ABZ to experimentally infected mice for five days did not increase the effectiveness of ABZ. Considering the larvae number obtained from the mice tissues, it was determined that rhizome extract in particular reduced the effect of ABZ. On the other hand, it was found noteworthy that the treatment efficiency of rhizome extract was higher when administered alone. The larvae number in the group given rhizome extract decreased as much as ABZ-treated group. The results obtained from this study shed light on future studies on the antiparasitic effects of *A. rupicola* Boiss *rupicola*, particularly the rhizome extract.

## Data Availability

No datasets were generated or analysed during the current study.
